# Interventions for Students’ Well-Being at the University of Helsinki (INSIGHT): Protocol and Preliminary Descriptive Results for a Quasi-Experimental Controlled Trial of a Social Identity Intervention and Two Active Comparators

**DOI:** 10.2196/79319

**Published:** 2026-01-23

**Authors:** Silja Martikainen, Oona Karhunen, Iiris Mankki, Vera Gergov, Päivi Berg, Satu Venäläinen, Elisa Rissanen, Johanna Lammintakanen, Erkki Heinonen, Anu Lehtinen, Janna Pöntinen, Henna Asikainen, Nina Katajavuori, Outi Linnaranta, Milla Räisänen, Samuli Salminen, Jari Lahti

**Affiliations:** 1 Department of Psychology, Faculty of Medicine, University of Helsinki, Haartmaninkatu 3, Helsinki, 00290, Finland, +358 294129841; 2 Department of Education, Faculty of Educational Sciences, University of Helsinki, Helsinki, Finland; 3 Inclusion and Equality Research Group, Faculty of Social Sciences, University of Helsinki, Helsinki, Finland; 4 Department of Health and Social Management, University of Eastern Finland, Kuopio, Finland; 5 Department of Psychology, University of Oslo, Oslo, Norway; 6 The Department of Healthcare and Social Welfare, Finnish Institute for Health and Welfare, Helsinki, Finland; 7 University Services, Sustainable Wellbeing Unit, University of Helsinki, Helsinki, Finland; 8 HYPE Centre for University Teaching and Learning, Wells Research Project, Faculty of Educational Sciences, University of Helsinki, Helsinki, Finland; 9 HYPE Centre for University Teaching and Learning, Faculty of Educational Sciences, University of Helsinki, Helsinki, Finland; 10 Folkhälsan Research Centre, Helsinki, Finland

**Keywords:** university students, group intervention, effectiveness, cost-effectiveness, wellbeing, social inclusion, equality, multimethod research, controlled study, groups 4 health

## Abstract

**Background:**

University students’ mental health problems are prevalent globally, which underlines the need for accessible and cost-effective mental health services in universities. Loneliness is a key risk factor for mental health problems, and it disproportionately affects students from minority backgrounds. Therefore, addressing loneliness and fostering inclusion and equality can be crucial strategies for enhancing students’ well-being.

**Objective:**

The aim of this study is to investigate a social-identity group intervention called Groups 4 Health (G4H) for university students’ well-being using both quantitative and qualitative methods. Here, we present the research protocol and report preliminary descriptive findings from the study cohort.

**Methods:**

The quantitative part of the study is a 4 parallel-arm nonrandomized controlled trial aiming to recruit 600 student participants from the University of Helsinki. The experimental group, which receives the G4H intervention, includes 5 group meetings held over a 7-week period. The experimental group will be compared with 2 active comparators: groups organized by the University of Helsinki study psychologists and a 7-week online intervention course focused on well-being and study skills, and to a no-intervention control group. The primary quantitative outcomes of the study are loneliness and depression; secondary outcomes include several measures of students’ well-being, academic performance, and cost-effectiveness of the intervention. Quantitative data are collected before the intervention, during the intervention (at week 3), immediately post intervention (at week 7 after baseline), and at 1- and 3-month follow-ups. The qualitative part of the study explores the challenges and opportunities related to inclusion and equality identified in the G4H intervention using observations, interviews, and focus group discussions.

**Results:**

In the preliminary findings based on the first data freeze in March 2025, we observed differences in the background characteristics between the trial arms, highlighting the need to address group selection bias. First results from the study are expected in 2026.

**Conclusions:**

If proven effective, these interventions have significant potential to improve students’ well-being in both short and long term, fostering mental health and supporting academic success and future career paths.

## Introduction

### Background

University students’ mental health problems are highly prevalent globally [1]. In Finland, 36% of university students reported high levels of psychological distress and 24% reported increased loneliness in the *2021 Finnish Student Health and Well-Being Survey* [2]. Anxiety and depressive symptoms at the start of university studies are often persistent and related to poorer academic performance and lower engagement [3]. The need for accessible, cost-effective mental health services in universities is substantial [4].

Loneliness (the discrepancy between one’s actual and desired social relationships) has been recognized as one of the key risk factors for numerous adverse psychological and somatic health outcomes among students and youth [5] and in various population-based samples [6,7].

Having positive relationships and feeling socially connected is also essential for academic performance. Loneliness, social isolation, and low social status impact intellectual achievement [8], whereas engaging in the academic environment reinforces individuals’ learning activities [9].

Mental health problems are more prevalent among many minority groups, who experience psychological symptoms more often than the rest of the population [10-12]. Several studies have also found that belonging to more than one minority group increases the risk of stressful life situations, mental health problems, and thus the need to use mental health services [13,14]. Belonging to minorities may also increase the risk of social isolation. According to the School Health Promotion study [12], young people belonging to gender and sexual minorities show elevated levels of loneliness, exclusion, and difficulties in receiving help in Finland. In addition, young people with immigrant backgrounds and young people with disabilities face considerable risks of loneliness [15]. These difficulties are largely caused by minority stress, which in turn has been linked with experiences of discrimination and unequal treatment [12,16].

With this background, interventions targeting loneliness and supporting social connectedness, inclusion, and equality could be a significant way to approach the student mental health crisis. The Groups 4 Health (G4H) intervention studied in this protocol aims at improving well-being by supporting individuals’ social group memberships that are compatible with their social identity [17].

G4H builds on the social identity approach to health (SIAH) [18,19] and the social identity model of identity change (SIMIC) [20,21]. The SIAH [18,19] comprises 2 related theories, the social identity theory [22] and self-categorization theory [23], claiming that individuals’ internalized group memberships have a significant role in their sense of self and that shared identities are important for productive social interaction. Therefore, belonging to multiple social groups that align with the individual’s identity is crucial for a sense of purpose and belonging, as well as for receiving support from others [18]. The role of group memberships is further emphasized during transitional life events such as moving to another country or entering a university, which may be stressful and may also affect an individual’s access to their social groups. The SIMIC further states that during stressful and transitional life situations, well-being can be enhanced by both managing and strengthening the individual’s social group memberships before the life change (the social identity continuity pathway) and by becoming a member of new social groups that align with the individual’s identity (the social identity gain pathway) [20,21].

The fact that G4H addresses major life changes and social identities makes it particularly relevant in the university context. Many students experience major changes, such as relocating to a new country or city, integrating into new communities, and leaving familiar ones behind. For minority students, finding new social groups that align with their identity can be even more challenging, emphasizing their need for support.

This protocol provides a unique opportunity to study the G4H in the university context with a multimethod approach that integrates both quantitative and qualitative methods. By implementing this approach, we aim to capture not only the effectiveness of the G4H intervention and the participant- or facilitator-related factors that moderate the effectiveness, but also the perspectives of minority students participating in the intervention, which is an aspect that has been overlooked in previous research. The inclusion of qualitative methods is crucial, as it allows us to gain deeper insights into the experiences and potential challenges faced by these students, enriching our understanding of the intervention’s impact.

Additionally, neither the cost-effectiveness of the intervention nor its effectiveness on academic performance has been explored in previous studies. Both of these analyses are essential for assessing its suitability and adaptability in promoting student well-being. Furthermore, to our knowledge, the effectiveness of the G4H has been assessed only in Australia in studies organized by the developers of the intervention. These represent clear gaps in the existing literature that this protocol aims to address.

### Aim and Objectives

The aim of this study is to investigate the effectiveness of the G4H intervention for university students’ well-being using both quantitative and qualitative methods. Our study includes 2 active comparators: intervention groups facilitated by the University of Helsinki (UH) study psychologists and an online intervention course focused on well-being and study skills. A control group of students who are not participating in any active well-being services at UH is also included.

Our first objective is to investigate the effectiveness and cost-effectiveness of the G4H intervention in (1) supporting well-being (most importantly by reducing loneliness and depression) and (2) supporting academic performance among university students. The second objective is to explore which factors moderate or mediate the effectiveness of the interventions. The third objective is to focus on the G4H intervention’s inclusiveness and the capacity of the G4H intervention to promote equality via qualitative methods.

Our research questions (RQs) are as follows:

- RQ1: How effective is the G4H intervention in reducing loneliness and depression and in supporting university students’ well-being compared with the 2 active comparators and the control group? (objective 1)
- RQ2: Does the G4H intervention support academic performance compared with the active comparators and the control group? (objective 1)
- RQ3: Is G4H cost-effective compared with the 2 active comparators and the control group? (objective 1)
- RQ4: How do factors related to the participant, facilitator, or intervention fidelity mediate or moderate the effectiveness of the intervention? (objective 2)
- RQ5: What are the challenges and opportunities related to inclusion and equality in the G4H intervention? (objective 3)

Compared with the no-intervention control group, we hypothesize that all active interventions reduce students’ loneliness and depression (RQ1) and improve students’ academic performance (RQ2). However, participants in the G4H intervention will experience a more long-term reduction in loneliness than those in the other active intervention groups [24]. We hypothesize that treatment alliance [25,26] and higher fidelity to the G4H manual mediate the interventions’ outcomes (RQ4). Regarding facilitators’ characteristics, we hypothesize that their greater self-rated skills and enjoyment in therapeutic work as well as agreeableness, openness, and engagement in personality traits and interpersonal relating will be positively associated with treatment alliances and intervention outcomes; and, conversely, greater difficulties and negative feelings in therapy work will be negatively associated with treatment alliances and outcomes [27-29]. Regarding motivation (RQ4), we set our hypotheses based on the self-determination theory [30] and the integral model of treatment motivation [31]. We hypothesize that autonomous motivation to participate in the intervention predicts greater engagement and increased effectiveness, whereas controlled motivation predicts lower engagement and effectiveness. We further hypothesize that engagement partially mediates the relationship between motivation and intervention effectiveness. No preliminary hypotheses are posed regarding RQs 3 and 5.

## Methods

### Trial Design

We conduct a parallel arm nonrandomized controlled trial with a 1:1 allocation ratio. We compare 3 intervention arms to a no-intervention control group as follows:

- Arm 1: The experimental group, G4H.
- Arm 2: Active comparator group 1, the study psychologists’ group interventions (SPGs)
- Arm 3: Active comparator group 2, toward better well-being and studying intervention (TBWS).
- Arm 4: No-intervention control group.

**Figure 1.**
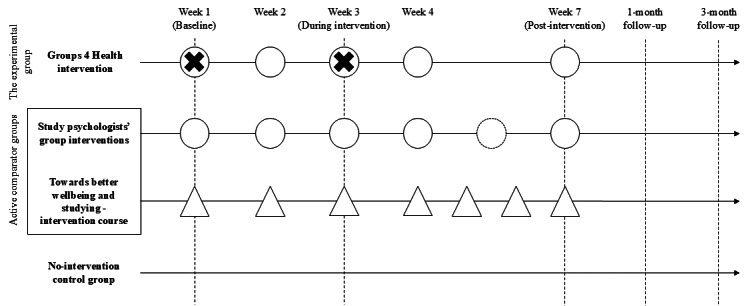
Trial arms, intervention schedule, and points of assessment. The dashed vertical lines indicate points of assessment. A circle with a dashed outline indicates an optional meeting. △: online meeting; ○: live or online meeting; X: Groups 4 Health facilitators’ assessments.

The study design and timeline are presented in Figure 1. The study follows the Standard Protocol Items: Recommendations for Interventional Trials (SPIRIT) guidelines.

### Interventions

#### Overview

The interventions in each active trial arm represent courses provided by UH for students who are seeking help to improve their overall well-being and study skills. Additionally, a control group consisting of students receiving no intervention is included. Participation in the study and in the interventions is voluntary, and participants can withdraw from participating at any point without any negative consequences.

#### Arm 1: G4H (The Experimental Group)

G4H is a manualized group intervention that targets social group memberships with the aim of improving well-being [17], as discussed in the introduction.

The G4H intervention consists of five 90-minute sessions that aim to provide participants with the knowledge and skills they need to effectively manage their social group memberships and identities. Each of the 5 intervention sessions pertains to 1 module. Each module integrates exercises and discussions from the G4H workbook targeting various aspects of group dynamics as outlined in the SIAH and SIMIC frameworks. In brief, the first module “schooling” provides psychoeducation about the importance of social group memberships for health; the second module “scoping” supports the participants in exploring their social identities and networks; the third module “sourcing” aims at strengthening the social identities that are valued by the participant; the fourth module “scaffolding” supports making a social action plan to establish new valued group memberships; and finally, the fifth module “sustaining” aims at revisiting the intervention, discussing any potential difficulties, and encouraging the maintenance of group memberships [17]. A detailed description of the modules can be found in the G4H manual [32].

At the UH, the G4H group size can vary between 4 and 9 university students. The groups are mostly organized face-to-face, although some groups meet entirely remotely. The students earn 2 European Credit Transfer and Accumulation System (ECTS) credits for completing the G4H intervention.

G4H groups at the UH are facilitated by 2 psychology students carefully trained for the intervention and mentored by experienced clinical psychologists. Psychology students interested in facilitating the G4H intervention can sign up for G4H facilitator training. During the 2-month training course, facilitators are introduced to the intervention literature (approximately 15 h) and attend lectures on the background theory, structure, and content of the intervention (approximately 6 h). In the more practical part of the training, the facilitators practice how to deliver the intervention and gain first-hand experience of participating in the intervention by conducting the intervention with each other. Finally, after the practice meetings, each pair facilitates one 5-session G4H intervention for a group of UH students. Additional reflection meetings are held with a clinically experienced psychologist (J Lahti), where the facilitators share their experiences and any problems or questions they may have encountered. During the training, facilitators keep a learning diary, which supports further introspection and reflection. The student facilitators earn 2.5 ECTS credits for completing the intervention training.

The effectiveness of G4H has been studied earlier in Australia. In a nonrandomized controlled study, G4H (n=83) significantly improved well-being and social connectedness of young adults after the intervention and in 6-month follow-up relative to the control group (n=75). In a randomized controlled trial by Haslam and colleagues [33], G4H (n=66) decreased loneliness and social anxiety and increased group memberships significantly more than treatment as usual (n=54) [17]. In a randomized noninferiority trial by Cruwys and colleagues [24], G4H (n=84) was compared with cognitive behavioral therapy (CBT; n=90). Both interventions were equally effective in reducing loneliness by the end of the intervention period. However, the G4H group demonstrated a maintained reduction in loneliness during the follow-up. Both interventions significantly reduced depressive symptoms, with no significant differences between G4H and CBT [24]. In all 3 controlled studies on the effects of G4H, the inclusion criteria were low mood and loneliness.

#### Arm 2: SPGs (Active Comparator 1)

The UH study psychology team organizes group interventions for various challenges related to studying for bachelor’s and master’s students. The SPGs offer client-centered peer support facilitated mostly by 2 study psychologists (some individual sessions may have only 1 facilitator). The recurring themes in the SPGs include challenges with attention, independent studying, scientific writing, and support for re-entering the studies after a break. The structure of the different thematic groups is similar—short presentations, group discussions, practices, and homework. These groups are implemented mostly as face-to-face meetings; however, in some groups, part of the meetings are conducted face-to-face and part as remote meetings. One group typically lasts for a study period and comprises five to six 90‐105 minute sessions. The typical group size ranges from 10 to 12 students. The interventions have potential in supporting study skills and the challenges of initiating study tasks, identifying and normalizing unpleasant thoughts and emotions related to studying, and acknowledging the role of one’s individual situation and well-being in study planning. The study psychologists’ work is bound by the legal and ethical requirements for licensed psychologists in Finland, overseen by the National Supervisory Authority for Welfare and Health (Valvira).

#### Arm 3: TBWS (Active Comparator 2)

TBWS is an optional, 7-week online intervention course available for all UH students that aims to foster well-being and study skills. The course protocol has been described in detail earlier by Asikainen and Katajavuori [34]. The course is based on the principles of acceptance and commitment therapy [35] and aims to promote psychological flexibility. In addition, it aims to support study skills such as time management. The course takes place on an online learning platform (Moodle), and the students earn 3 ECTS credits for completing it. This course progresses weekly and has 6 central themes, including an introduction to the themes, individual work in practicing psychological flexibility and study skills, and weekly peer group discussions. The peer group work is an important part of the intervention and lasts throughout the course. In the peer groups, there are about 5 students in each group, and the groups remain the same throughout the course. Acceptance and commitment therapy–based interventions have been shown to support well-being among university students [36], and previous studies have suggested that this course can improve students’ well-being and studying in several ways [37,38]. Thus, participating students have the potential to benefit from this intervention in terms of increased well-being.

#### Arm 4: The No-Intervention Control Group

The control group consists of students who are currently not taking part in any group interventions for study skills or psychological well-being at the UH. The participants in the control group may, however, take part in other social and mental health services than those provided by the UH, and this is not restricted during their participation in the study. The recruitment and data collection among the control group are scheduled to take place simultaneously with the active intervention groups: the participants are recruited at the same time, and they answer the questionnaires at the same pace as the active interventions progress during the academic year.

The interventions studied in this protocol have been developed for educational contexts and thus are expected to be well-suited for the university context. As discussed earlier, the G4H builds on SIAH and SIMIC, particularly aiming at supporting well-being during transitions in life, such as entering a university [21]. In addition, both the SPGs and the TBWS courses have been specifically developed by experts within the UH for supporting university students’ well-being and academic ability.

### Participants

#### Setting and Timeline

The study is conducted in an academic setting among UH students in Finland. The recruitment has progressed as follows: for the RQ1-RQ3, the recruitment for the first data freeze ended at the end of 2024, and for RQ5, the recruitment ends by the end of the year 2025. For the more explorative objective 2 (RQ4) on the factors that mediate or moderate intervention effectiveness, we assume that a larger sample is required, and recruitment continues longer (current ethical approval allows data collection until the end of 2028). We completed the first data freeze in March 2025 for the RQ1 to RQ3 and will complete the data collection of the qualitative data for the RQ5 by December 2026.

The quantitative data collection started in March 2022 for the G4H and the SPGs. From October 2022, the data collection from all 3 interventions and the no-intervention control group has taken place simultaneously. The intervention schedule and points of assessment are illustrated in Figure 1. It should be noted that data on health care and social service use, as well as the participants’ minority status and readiness to attend the intervention, were gathered only from students who participated starting from March 2023 for the no-intervention control group and from September 2023 for the active intervention arms. The research ethics committee was informed about these added outcome measures, and a new ethical approval was obtained from the committee before data collection started.

In the collection of qualitative data, participant observation is conducted in the meetings of the G4H group and interviews with facilitators (n=9) and intervention participants (n=11) at the end of the intervention period. In addition, the follow-up interviews with intervention participants will be conducted 12 months after the first interview (2024‐2026). Additionally, focus groups with young people belonging to minorities (n=19 participants) are arranged to discuss G4H in terms of equality and inclusion during the data collection period (2023‐2025).

#### Sample Size

For RQ1-RQ3, the target minimum sample for the quantitative study is 600 participants, distributed evenly across 4 groups (150 participants per group). Since comparable previous studies with a similar group of participants using the same outcome measures are not available, it was not possible to make exact a priori sample size calculations. However, based on an estimation with the G*Power software (version 3.1) [39] on a repeated measures analysis of variance design with between-groups factors, this target sample size would yield at least 95% power for the detection of small effect sizes (*f*=0.20) as defined by Cohen [40] when considering multiple comparisons and a 35% attrition rate. Previous studies with different designs investigating the G4H intervention have demonstrated significant treatment effects with smaller samples ranging from 66 [33] to 84 [24] participants in the G4H group, supporting the expectation that our larger sample will be sufficient to detect meaningful treatment effects, or lack thereof.

#### Recruitment

The UH student well-being services are available to all students at the university, and there are no exclusion criteria for the study. All the students willing to participate are included regardless of their level of loneliness, state of psychological well-being, or stage of studies. Students are recruited for the active intervention groups and no-intervention group by email, website, and social media advertisements circulated by the UH media and communications services, communications services at the faculties, student health services, and student organizations. The students are not randomly assigned to the intervention groups and can sign up for a course of their choice. The facilitators of the G4H intervention are also invited to participate in the study during the G4H facilitator training. All facilitators willing to participate are included in the study. Taking part in relevant concomitant care is not prohibited during the intervention study. The participating students or facilitators are not blinded to the study protocol.

### Quantitative Methods

To collect the quantitative data, we use paper-and-pencil and electronic versions of the questionnaires. Those in the active intervention groups fill out the questionnaires before or after the intervention sessions if the questionnaires are provided electronically and during the session if the questionnaires are provided on paper. Those in the no-intervention group fill out the questionnaires based on the email reminders of the electronic data collection system (time interval between reminders is the same as the interval between sessions in the active intervention groups).

Electronic data collection is implemented in the Research Electronic Data Capture (REDCap) system (Vanderbilt University) [41], provided by the UH for collecting sensitive research data electronically. In the case of the TBWS intervention, data collection from time points before and immediately after the intervention is partly integrated into the online course platform, and the students can respond either via the course platform or via the REDCap system, while data collection from week 3 and the 1- and 3-month follow-ups is implemented only in the REDCap.

Before the analysis phase, the data validity is confirmed via range checks, missing value checks, and outlier detection. Potentially erroneous data are checked against the paper and electronic forms, and other necessary sources, and corrected if needed. All modifications are documented.

Participant retention is promoted by reminding the participants during the group meetings to fill out the questionnaires. All participants receive up to 5 reminder messages from the electronic data collection system if they do not complete the necessary questionnaires on time. Retention is also promoted by the possibility of receiving written feedback on the questionnaire results after participation. Participating students also receive movie tickets as compensation for completing all follow-up questionnaires. Adherence to the G4H intervention is monitored by documenting the number of sessions in which each student participated.

All the quantitative methods used are presented in Table 1, which also includes the measurement time points and study arms from which the data are collected. Multimedia Appendix 1 includes a table that describes the unabbreviated titles of each scale, scale details, and information on their reliability and validity. Most measures have demonstrated at least acceptable internal consistency (Cronbach α≥0.70) and convergent validity with other similar measures. The few exceptions with lower Cronbach α are either very short scales (4 items) or include a specific subscale that performs poorly.

**Table 1.** The quantitative assessment design including areas of assessment, used measures, and an assessment schedule

Assessment	Scales, forms, and measures	Time points and arms (1-4)^a^ where data are collected						
		Week 1	Week 2	Week 3	Week 4	Week 7	1-month follow-up	3-month follow-up
Primary outcome measures								
Loneliness	ULS-8^b^ [42]	1‐4	—	—	—	1‐4	1‐4	1‐4
Loneliness	ULS-4^c^ [43]	1‐4	—	1‐4	—	1‐4	1‐4	1‐4
Depression	PHQ-9^d^ [44]	1‐4	—	—	—	1‐4	1‐4	1‐4
Secondary outcome measures								
Active group memberships	EXITS^e^ [45]	1‐4	—	1‐4	—	1‐4	1‐4	1‐4
Mental well-being	SWEMWBS^f^ [46]	1‐4	—	—	—	1‐4	1‐4	1‐4
General well-being	GP-CORE^g^ [47]	1‐4	—	—	—	1‐4	1‐4	1‐4
Anxiety	GAD-7^h^ [48]	1‐4	—	—	—	1‐4	1‐4	1‐4
Social anxiety	Mini-SPIN^i^ [49]	1‐4	—	—	—	1‐4	1‐4	1‐4
Quality of life	EQ-5D-5L^j^ [50]	1‐4	—	—	—	1‐4	—	1‐4
Use of social and health care services	Health service usage self-report (medications, health care appointments, hospitalization, and counseling)	1‐4	—	—	—	1‐4	—	1‐4
Time used by facilitators	Time used at planning, executing, and assessing the sessions	1	1	1	1	1	—	—
Study credits	UH^k^ registries, 1 year before and 1 year after the intervention (arms 1‐4)	—	—	—	—	—	—	—
Grade point average	UH registries, 1 year before and 1 year after the intervention (arms 1‐4)	—	—	—	—	—	—	—
Weighted grade point average	UH registries, 1 year before and 1 year after the intervention (arms 1‐4)	—	—	—	—	—	—	—
Other outcome measures								
Identification with the intervention group	SISI^l^ [51]	—	—	1‐3	—	—	—	—
Participants’ working alliance	WAI-SR^m^ [52]	—	—	1‐2	—	—	—	—
Facilitators’ working alliance	WAI-SRT^n^ [53]	—	—	1	—	—	—	—
Mood	Analog mood scale (0‐7)	1‐4		1‐4		1‐4	1‐4	1‐4
Fidelity to the G4H manual	G4H^o^ manual fidelity checklist	1	1	1	1	1	—	—
Participant attendance	Number of sessions participated	1	1	1	1	1	—	—
Background variables								
Participant background								
Sociodemographic characteristics	Gender, birth date, mother tongue, marital status, household composition, residential situation, employment status, education, income, subjective financial situation, subjective health, subjective loneliness, and subjective minority status	1-4	—	—	—	—	—	—
Social support	SSQ3^p^ [54]	1‐4	—	—	—	—	—	—
Treatment motivation	SIMS^q^ [55], tailored motivation rulers [56]	1‐3	—	—	—	—	—	—
Organized studying	4 items from a HowULearn questionnaire [57]	1‐4	—	—	—	—	—	—
Facilitators’ assessment								
Sociodemographic characteristics	Gender, birth date, mother tongue, marital status, household composition, residential situation, employment status, education, and income	—	—	—	—	—	—	—
Professional and individual characteristics	XS5^r^ [58], ECR-S^s^ [59], TBIF-PI^t^ [60]	1	—	—	—	—	—	—
Self-assessed skillfulness	TWIS^u^ [61]	—	—	1	—	—	—	—

a Arm 1: Groups 4 Health intervention; arm 2: study psychologists’ group interventions; arm 3: toward better well-being and studying intervention; and arm 4: mo-intervention control group.

b ULS-8: 8-item University of California Los Angeles Loneliness Scale.

c ULS-4: 4-item University of California Los Angeles Loneliness Scale.

d PHQ-9: Patient Health Questionnaire.

e EXITS: Exeter Identity Transition Scales

f SWEMWBS: Short Warwick-Edinburgh Mental Well-being Scale.

g GP-CORE: General Population version of the Clinical Outcomes in Routine Evaluation –scale

h GAD-7: Generalized Anxiety Disorder -questionnaire.

i Mini-SPIN: Mini Social Phobia Inventory.

j EQ-5D-5L: EuroQol - 5 dimension - 5 level questionnaire.

k UH: University of Helsinki.

l SISI: Single-Item Social Identification measure.

m WAI-SR: Working Alliance Inventory Short and Revised.

n WAI-SRT: Working Alliance Inventory Short and Revised Therapist version.

o G4H: Groups 4 Health.

p SSQ3: Social Support Questionnaire (three-item version).

q SIMS: Situational Motivation Scale.

r XS5: Extra-Short Five.

s ECR-S: Experiences in Close Relationships Short form.

t TBIF-PI: Personal Identity scale of the Trainee Background Information Form.

u TWIS: Therapist Work Involvement Scales.

#### Primary Outcomes

The primary outcomes are loneliness and depression (refer to Table 1 and Multimedia Appendix 1 for details). Based on previous evidence, the G4H intervention has at least equal potential to promote well-being in these areas as CBT [24].

#### Secondary Outcomes

The secondary outcomes are active group memberships, mental well-being, general well-being, anxiety, social anxiety, academic performance, health-related quality of life, use of social and health care services, intervention costs, and cost-effectiveness of the intervention (refer to Table 1 and Multimedia Appendix 1 for details).

To assess participants’ academic performance, data will be retrieved from the UH student register (Sisu student information system). Academic performance is determined by 3 outcome variables: overall accumulated study credits, the grade point average (GPA) of the study credits and the weighted GPA. When determining the weighted GPA, only completed studies that have been assessed on a numeric scale (from 1 to 5) are considered, as some courses accumulate study credits but are graded only as passed or failed. Weighted GPA is determined by the grades of each course weighted by the number of credits gained for it. The academic performance data are collected from 2 time periods: a 1-year period before and a 1-year period after starting the assigned intervention (baseline, week 1).

Cost-effectiveness analyses will apply a societal perspective by including intervention costs as well as university students’ health and social services costs. For the cost-effectiveness analyses, the facilitators will provide information on the time used for planning, executing, and assessing the sessions to evaluate the costs of the G4H intervention. Furthermore, the students in all trial arms fill out 2 questionnaires before the intervention, immediately after the intervention, and at 3-month follow-up (Table 1). The questionnaires measure health-related quality of life and self-reported use of health and social services (Table 1). The use of health and social services will be monetarized using Finnish national unit costs [62].

#### Other Outcomes

Other outcome measures include the participant’s identification with the intervention group, attendance, and mood, as well as facilitators’ fidelity to the intervention manual, and both the participant’s and facilitator’s evaluations of the working alliance (Table 1 and Multimedia Appendix 1). These measures are used to examine their potential in moderating or mediating the effectiveness of the intervention. Fidelity to the intervention manual is measured with a questionnaire detailing how well the facilitators covered the core themes of each intervention session. Each facilitator pair fills in the questionnaire immediately after each intervention session. This questionnaire is the same as used by the previous study that assessed program adherence to the G4H [33] .

#### Background Variables

In addition to the outcome measures described above, survey data on the participants’ background are gathered to account for them in the analyses and to examine their potential in moderating or mediating the effectiveness of the intervention (Table 1 and Multimedia Appendix 1). The participant background questionnaire includes questions on sociodemographic characteristics (refer to Table 1 for details), experienced social support, organized studying, and situational intrinsic and extrinsic motivation. Readiness to attend the intervention is enquired by a set of readiness rulers adapted from Miller and Rollnick [56]. Furthermore, data are gathered from the UH registries, including information on study rights (faculty, degree programs, and fields of study) and their starting years, active and passive registration, and completed degrees.

#### Facilitators’ Characteristics

To investigate how facilitators’ individual professional and personal characteristics may predict the intervention process and effectiveness, G4H facilitators complete self-report measures both before and during the interventions (Table 1 and Multimedia Appendix 1). Before initiating the intervention, facilitators complete questionnaires related to personality traits, attachment style, and self-experiences in close personal relationships. After the third session, the facilitators report experiences of their current skillfulness, difficulties, and emotions in group work.

### Statistical Analyses

We will conduct our analysis following the intention-to-treat principle, including all participants regardless of whether they completed the intervention.

The change in the outcome variables over time will be analyzed with linear mixed-model analyses due to the hierarchical data structure. Information criteria are used to assess model fit and determine the appropriate model structure. Mixed effects models can also be fitted for participants with missing data from one or more time points. If needed, other appropriate imputation methods, such as multivariate imputation by chained equations [63], will be used. For this reason, the number of participants included in the analyses is likely to be higher than the number of participants providing data at the last follow-up.

We will explore with logistic regression analyses whether background characteristics and baseline psychiatric symptoms predict study dropout, and necessary means to account for any systematically missing data will be taken. We aim to show that the missing observations of the outcome variable, due to dropout, are conditionally independent from the observed values of the outcome variable given our model for dropping out from the study. If needed, inverse propensity score weighting is carried out so that the treatment effects are conditionally independent from the treatment group assignments and from differing dropout rates given the propensity scores. We consider individuals’ selection into different study groups (arms) under the stable unit treatment value assumption based on their background characteristics, given that we have access to an extensive dataset. The selection of individuals into groups is accounted for by making the estimators double robust, which is a well-known approach to adjust for selection into groups based on background characteristics in model-based estimates [64,65]. This ensures that our results are unbiased and consistent estimators of the treatment effects. Potential effects of the COVID-19 pandemic on the stability and change in students’ well-being will be inspected and considered in the analyses if necessary.

All models will be inspected for normality of residuals, homoscedasticity, and multicollinearity. The outcome variables will be transformed to attain normality if needed. Appropriate interaction terms are added for further subgroup analyses. Potential mediator (eg, therapeutic alliance, motivation, and model fidelity) and moderator (eg, participant and facilitators' background) effects will be inspected in additional mediation and moderation analyses.

In the case of missing questionnaire items, within-person imputation is carried out for responses when less than 50% of the answers are missing per scale (the within-person average of the existing items is imputed to the missing responses). We acknowledge that this procedure may decrease the within-person variation in the data. However, as we discuss later, the prevalence of item nonresponse in the dataset is low.

We will take appropriate measures to balance between Type I and Type II error in the reporting of analyses results. If needed, we will use methods to account for multiple testing such as false discovery rate or new eigenvalue-based methods that provide a more accurate adjustment for nonindependence among tests [66].

### Cost-Effectiveness

We will conduct 3-month follow-up cost-utility and cost-effectiveness analyses. The cost-utility analysis will use EQ-5D-5L–based quality-adjusted life-years as the effectiveness outcome measure [50,67]. The cost-effectiveness analysis will use both primary outcomes of the effectiveness study, namely loneliness measured with the 8-item University of California Los Angeles Loneliness Scale [42] and depression measured with the Patient Health Questionnaire [44] (Table 1 and Multimedia Appendix 1) as the effectiveness outcome measure. The analyses will use a societal perspective by including the intervention costs and costs of students’ health and social service use. The analyses will be conducted with the intention-to-treat principle. The missing outcome measures will be imputed with multiple imputation methods [68]. We will estimate incremental net health benefits between interventions. The estimation of the incremental net health benefits and 95% CIs will be used for statistical methods (eg, ordinary least squares regressions) [69]. We will perform multiple 1-way sensitivity analyses to test the robustness of the economic evaluation results. The results of sensitivity analyses will be presented in a tornado diagram. If interventions show effectiveness, we will conduct decision-analytical modeling of long-term cost-utility and cost-effectiveness beyond 3-month follow-up data.

### Qualitative Methods

We explore inclusion and equality, with respect to G4H using three qualitative data collection methods: (1) participant observation, (2) interviews with G4H participants and facilitators, and (3) focus groups with young people who belong to minorities. The aim is to examine challenges and possibilities in the G4H intervention regarding social inclusion and equality and whether G4H promotes long-term social inclusion and equality among the participants. The focus groups target young people belonging to sexual and gender minorities, young people with immigrant backgrounds, and young people with disabilities. These groups represent youth minorities with a considerable risk of loneliness [15]. To date, research on their experiences with different services is lacking [12,70-72]. These groups are also underrepresented in higher education [2,73,74].

The participant observation focuses on the interaction and the practices undertaken in the groups to gain knowledge about how the groups work, what kinds of issues they deal with, and especially how inclusion and equality are accomplished in the groups (are there minority participants, can participants influence the themes dealt with in the groups, or is inclusion and equality taken into account in some other ways in the groups). Following the ethnographic research tradition, the researcher takes part in the activities of the groups instead of observing them from the outside. Depending on their preferences, the study participants (both facilitators and participants in the groups) will be interviewed in a group, in pairs, or individually, in facilities located outside the university (eg, in a study room of a local library).

In the focus groups, young people belonging to minorities are asked to comment on the design or description of G4H in terms of equality and inclusion, with the goal of enhancing inclusivity by listening to their views on the interventions and accumulating knowledge on the fit between the interventions and their specific needs. All the interviews will be tape-recorded and transcribed for analysis.

To ensure the validity of qualitative findings, data triangulation is used via collecting 3 different types of data: focus group interviews, intervention participant interviews, and facilitator interviews. Setting these different data into a dialogue enables a multifaceted understanding of intervention effectiveness, inclusion, and equity. The analyses of qualitative data draw on minority stress theory [75] and intersectional theory [76,77].

The analyses are conducted by following reflexive thematic analysis [78,79]. Reflexive thematic analysis is conducted by following 6 steps that move from familiarization with data, coding, formulating, and refining themes to writing the report in alignment with the key principles of the approach, highlighting reflexivity and in-depth attention to nuance in participants’ meaning-making. If several analysts are involved in the analysis, the goal is to engage in discussion throughout each step of the analysis, to refine the analysis with the help of various analysts’ viewpoints. This approach, therefore, acknowledges that analysts’ interpretations of the data may differ, which is why transparency and a close consideration of the subjectivity of interpretation are highlighted. The first steps of the analysis may be conducted with the help of software (such as ATLAS.ti 24; Lumivero), but the actual analysis requires repeated rounds of reading of the whole dataset by the analysts. The emphasis on researcher reflexivity in this analytical approach further enhances the validity of interpretations and helps ensure that the findings reflect the multiplicity of participants’ viewpoints and accurately portray repetitive patterns in the data.

### Ethical Considerations

#### Overview

The study protocol was approved by the Research Ethics Committee in the Humanities and Social and Behavioural Sciences at the UH, Finland (Statements: 15/2022, 32/2023, 10/2025). The ethical board will be informed in case of important protocol modifications. Written informed consent to participate in the study is obtained from all participants and facilitators. The participants are informed about the study via an information sheet and a data protection notice detailing the research protocol, data management, and participants’ rights following the General Data Protection Regulation (GDPR) of the European Union. The consent form can be signed electronically or on paper. Electronic consent forms are delivered via an email from the REDCap system before the first intervention session or at a corresponding time point for the control group. Paper consent forms are delivered during the first meeting of each active intervention. The participants are also told that they may be contacted about potential ancillary studies. The participants are informed that they can withdraw from the study at any point without any consequences. The participants receive movie tickets as compensation for participating. A data monitoring committee has not been appointed as there is no suspicion of harm caused by the interventions studied in this trial to the participants.

#### Privacy and Confidentiality

The access to the data collection systems is restricted to assigned data managers. The questionnaire data collected on paper are transferred to REDCap by a data manager. Paper data are stored in a secure locked facility with restricted access provided by the Faculty of Medicine at the UH. All the data are downloaded from the data collection system and backed up on secure UH servers with restricted and password-protected access. Upon download, the data are pseudonymized via ID codes, and data that include personal identifiers are stored separately. All identifiers enabling linkage to the university records are removed from the data during the pseudonymization process. No such data that could risk reidentification of the participants is shared outside the research group.

The participants’ identifiers are not shared outside the research group at the UH. Other pseudonymized research data may be shared with selected research partners for further research. A research collaboration agreement and a data sharing and processing agreement, which specifies the secure management and use of the data, will be made.

The consent form including identifiers will be destroyed 10 years after the end of the research project. Pseudonymized data will be kept for later, compatible scientific research in accordance with the requirements of the GDPR. The data will be stored at a secure UH server and kept protected so that only those who need the data can access them. Before the data can be used for later, compatible scientific research, the controller will ensure that the new use of data is compatible with the initial purpose in accordance with the requirements of the GDPR. Participants will receive a new data protection notice on the new use of the research data, unless the controller can no longer identify the participants from the data.

#### Participants’ safety

We will do everything we can to ensure the safety of the participants. We do recognize that some topics discussed during the sessions, such as challenging group memberships and difficulties related to academic stress, may cause distress (by activating existing anxieties). The group sessions are designed to create a supportive environment where students feel secure and comfortable to express concerns to the facilitators. Each intervention has clear protocols on providing further support to the students if needed, and the training of the facilitators or course organizers includes plans to mitigate risks to the safety of the participants. Moreover, anonymous feedback is collected after all the interventions to tackle potential issues for the subsequent courses.

In the G4H course, potential challenges are addressed during the facilitator training and the mentoring sessions. Facilitators are instructed to consult with the course supervisor, J. Lahti, a clinical psychologist and licensed trainer-level psychotherapist, if they have any concerns about a participant’s well-being. Additionally, they are provided with resources for further support, including contacts within the university’s counseling services and public health care, which can be shared with participants if needed.

In the TBWS course, students are also provided resources for further support, including contacts within the university’s counseling services and public health care. After assessing their well-being in the beginning of the course, all students get feedback and are instructed to seek this additional support if they get worried about their well-being. In addition, students get detailed instructions for peer group discussions instructing them to focus on the course themes and how the learned aspects can support their studying rather than discussing their personal health issues. Students are also requested to contact course teachers if they become concerned about their own or a peer’s well-being.

In the SPGs, psychological safety is promoted by discussing the group’s rules during the initial meeting. Students have the option to anonymously inform the facilitators, via a note, about any specific concerns or needs they would like the facilitators to be aware of. Study psychologists guide group discussions and intervene in potential challenging situations. If concerns arise, the psychologist offers discussion support to the student. Additionally, students have the opportunity for individual guidance from the study psychologist, who can assist them in contacting health care services if necessary. Students are encouraged to provide anonymous feedback to identify any potential weaknesses of the intervention.

We are aware that the young people invited to participate in the study about inclusion and equality might be in vulnerable positions. Our research team has extensive experience in conducting qualitative interviews with young people in vulnerable positions or who belong to minorities, such as LGBTIQ+ young people (lesbian, gay, bisexual, transgender, intersex, queer, and other), young people with diverse ethnic backgrounds, and young people not in employment, education, or training. The interviewers aim at sensitivity in their interaction with the interviewees and, for instance, provide the interviewees with room for regulating the depth and detail in which they discuss their experiences and emotions. The interviews and focus groups have been carefully designed to avoid placing excessive mental strain on participants. Participants are free to decide for themselves how much detail they wish to share about their own experiences and what they wish to reveal about their minority identities and related experiences. In addition, the use of peer facilitators in the focus groups promotes a sense of security and confidentiality, as well as peer support.

The opportunity to talk about one’s experiences with the G4H intervention and the opportunity to be respectfully heard can be empowering for the interviewed young people. In addition, sharing one’s thoughts and experiences of observed practices can alleviate stress and provide a sense of social support. On the other hand, talking about potential negative aspects, or remembering and describing possible experiences and observations of discrimination, or talking about experiencing limitations related to gender identity, sexual orientation, ethnicity, or ability can cause minor, short-term discomfort. However, according to our evaluation, this is highly unlikely to exceed the discomfort caused by the challenges and small annoyances of normal everyday life. The participants will be offered an opportunity to talk about the feelings caused by the interview afterwards with the interviewer or the researcher responsible for the entire research project. They will also be provided with contact information for services that provide support for young people, and if necessary, the interviewer will help them find relevant services in their hometown. If the young person wishes to, they will be given a chance to read the transcript of the interview, that is, the transcribed text of the audio recording.

## Results

### Study Status

**Figure 2.**
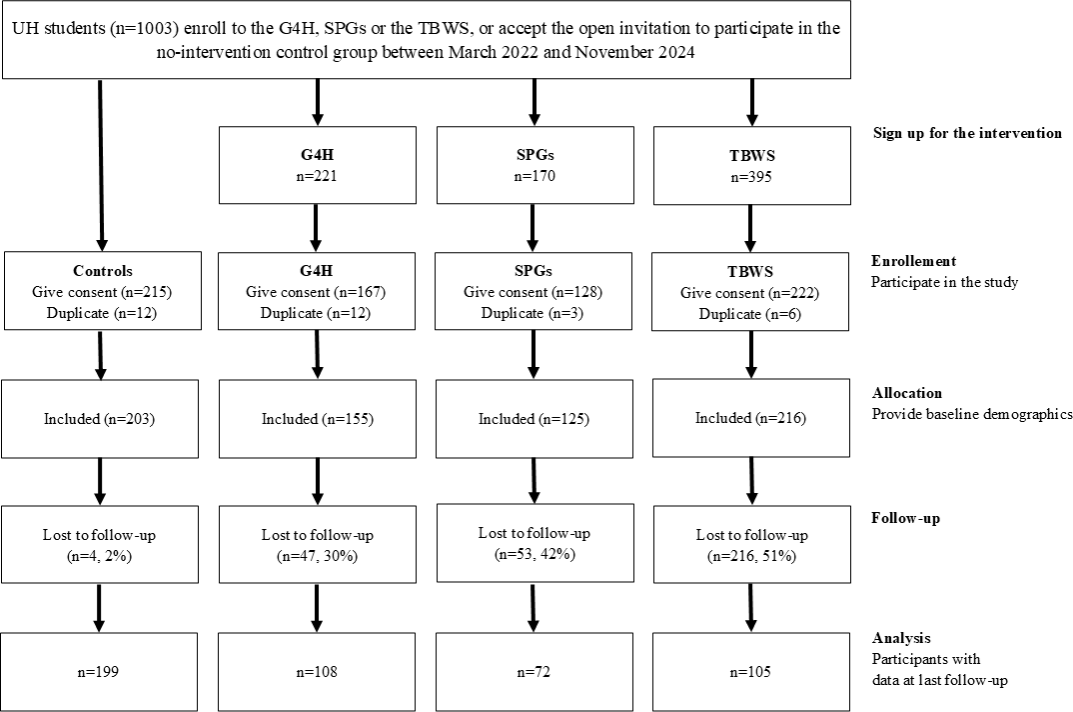
Study recruitment flow chart. Duplicate participants had already participated in the study (either in the same trial arm or in another arm). Controls: no-intervention control group; G4H: Groups 4 Health; SPG: study psychologists’ group interventions; TBWS: toward better well-being and studying intervention; UH: University of Helsinki.

Figure 2 illustrates the number of participants from the start of the study in March 2022 until the first data freeze in March 2025.

A high proportion of the university students enrolled in the interventions decided to participate in the study. The minimum target sample size (n=150) was attained for the G4H (n=155), TBWS (n=216), and control group (n=203). For the SPGs, the target sample size was not attained (n=125).

The anticipated study completion date for all data collection is by the end of the year 2028. Analyses for RQ1 and RQ3 are anticipated to take place in years 2025 and 2026, analyses for RQ2 in years 2026 to 2027, and analyses for RQ4 and RQ5 in years 2026 to 2028. First results from the study are anticipated to be published in the year 2026.

### Attrition

**Figure 3.**
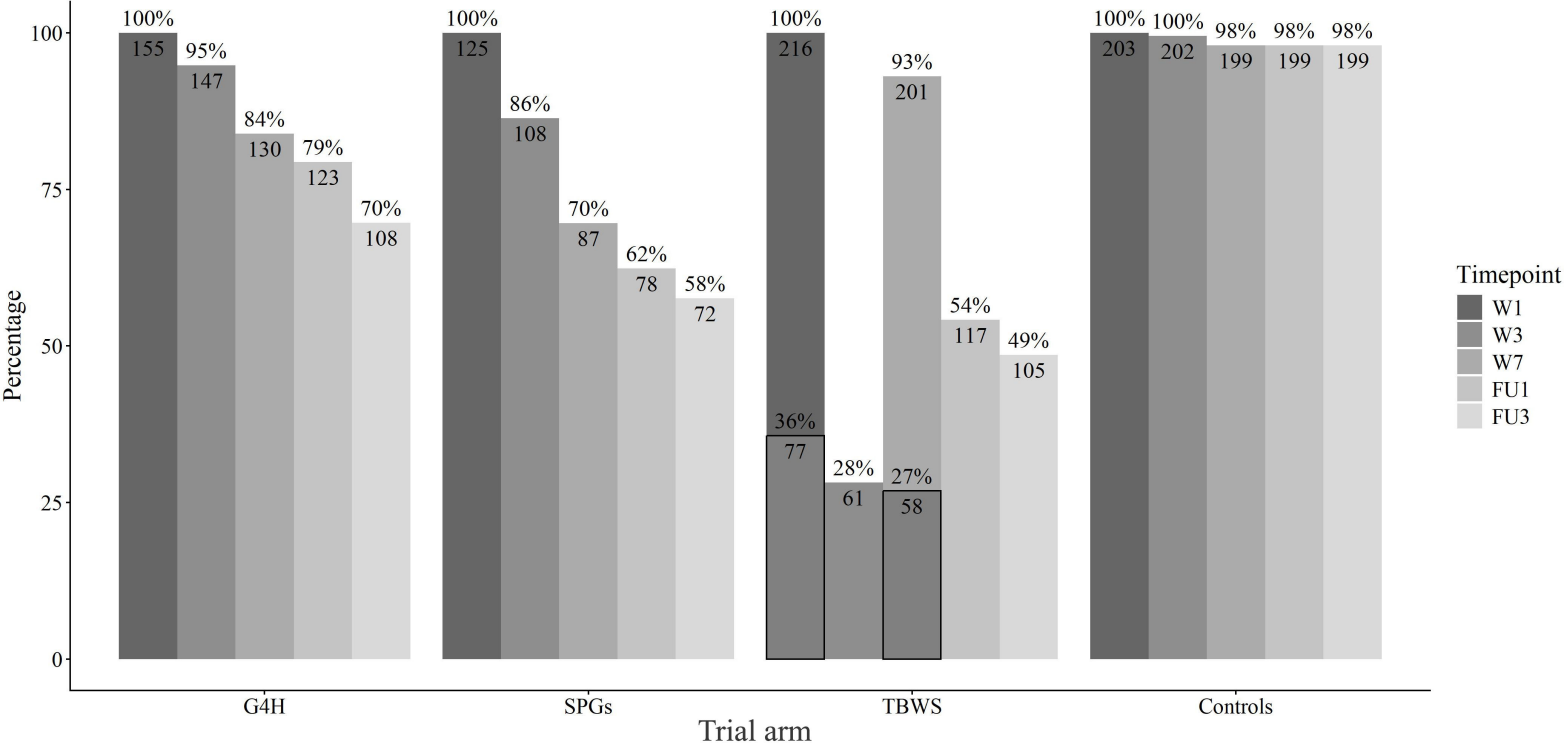
Attrition rates by trial arms. For TBWS, the smaller percentages at W1 and W7 denote the percentage of answers collected via REDCap. FU1: 1-month follow-up; FU3: 3-month follow-up; G4H: Groups 4 Health, SPG: Study psychologists’ group intervention; TBWS: toward better well-being and studying intervention, Controls: no-intervention control group, UH: University of Helsinki; W1: week 1; W3: week 3; W7: week 7.

Figure 3 illustrates the attrition rates by trial arms by the first data freeze in March 2025. The percentages do not accurately indicate the number of study dropouts, as all surveys are sent to participants regardless of nonresponse to previous surveys, allowing them to re-enter the study. The attrition rate has exceeded the anticipated 35% in the SPGs and TBWS. For the TBWS, the bars with black outlines represent the responses to the REDCap survey. The low response rate at the week 3 assessment (during intervention) is due to the survey only being available in REDCap and not integrated into the online course platform as in week 1 and week 7. Based on the attrition rate, we took action to increase retention, including adding incentives for the participants (a movie ticket for completing the surveys).

### Participant Descriptive Statistics

Table 2 illustrates the participants’ characteristics of the current sample at baseline by trial arm. In all groups, most participants are women (87/125, 70%‐175/203, 86%), not in a relationship (119/214, 56%‐107/154, 69%), and childless (175/208, 84%‐101/113, 89%). The age medians are lower than the age means (median_G4H_ 25, IQR 22-29 y, median_SPGs_ 27, IQR 25-31 y, median_TBWS_ 24, IQR 22-31 y, and median_Control_ 23 y, IQR 21-27 y).

**Table 2.** Characteristics of the study sample (n= 699) recruited between March 2022 and November 2024.

Characteristics	Trial arm				
	G4H^a^ (n=155)	SPGs^b^ (n=125)	TBWS^c^ (n=216)	Controls^d^ (n=203)	*P* value^e^
Age, mean (SD; range)	27.4 (8.0; 19-59)	28.9 (7.1; 20-53)	26.8 (7.0; 19-59)	25.1 (6.6; 18-58)	<.001
Missing, n	1	0	0	1	
Gender, n (%)					<.001
Woman	110 (71)	87 (70)	172 (80)	175 (86)	
Man	35 (23)	28 (22)	42 (19)	21 (10)	
Other	9 (6)	10 (8)	2 (1)	7 (3)	
Missing, n	1	0	0	0	
Relationship status, n (%)					.01
Single	107 (69)	78 (63)	119 (56)	130 (64)	
In a relationship	41 (27)	39 (31)	92 (43)	68 (33)	
Divorced or widowed	6 (4)	7 (6)	3 (1)	5 (2)	
Missing, n	1	1	2	0	
Living situation, n (%)					.05
Lives alone	79 (51)	57 (46)	86 (40)	95 (47)	
Lives with family	52 (34)	52 (42)	105 (49)	92 (46)	
Lives with others	23 (15)	16 (13)	23 (11)	15 (7)	
Missing, n	1	0	2	1	
Number of children, n (%)					.54
0	98 (88)	101 (89)	175 (84)	132 (88)	
≥1	14 (13)	12 (11)	33 (16)	18 (12)	
Missing, n	43	12	8	53	
Employment, n (%)					.08
Unemployed	87 (57)	58 (46)	113 (53)	90 (45)	
Employed	66 (43)	67 (54)	99 (47)	112 (55)	
Missing, n	2	0	4	1	
Gross income, n (%)					.07
<9 k €/year^f^	85 (56)	57 (46)	113 (55)	125 (62)	
9–‐18 k €/year^f^	40 (26)	46 (37)	51 (25)	49 (24)	
>18 k €/year^f^	28 (18)	21 (17)	43 (21)	29 (14)	
Missing, n	2	1	9	0	
Study years in current degree, mean (SD; range)	1.9 (1.6; 0‐8)	4.6 (3.7; 0‐23)	1.6 (2.3; 0‐19)	1.8 (1.8; 0‐10)	<.001
Missing, n	6	2	11	0	

a G4H: Groups 4 Health.

b SPGs: study psychologists’ group interventions.

c TBWS: toward better well-being and studying intervention.

d Controls: no-intervention control group.

e *P* values are from tests comparing the trial arms with each other, using Kruskal-Wallis tests with continuous variables and *χ*2 tests with categorical variables.

f 1 €=US $1.08 in March 2025.

To determine whether the participants in the trial arms differed from one another in terms of background characteristics, we performed chi-square tests for categorical variables and Kruskal-Wallis tests for continuous variables (based on visual inspection, neither followed a normal distribution) (Table 2). Trial arms differed in age (Kruskal-Wallis *χ*^2^_3_=40.7, *P*<.001), gender (*χ*^2^_6_=5.4, *P*<.001), relationship status (*χ*^2^_6_=15.8, *P*=.02), living situation (*χ*^2^_6_=12.6, *P*=.05), and study years in current degree (Kruskal-Wallis *χ*^2^_3_=125.7, *P*<.001). These observations highlight the need to address group selection bias.

### Missing Values Analyses

Item nonresponse (ie, a participant does not respond to an item within a questionnaire) was observed with missing data pattern plots across all groups. First, we examined the outcome variables which are assessed at all time points except week 3: loneliness, depression, mental well-being, general well-being, anxiety, social anxiety, multiple group memberships, and mood. Structural missingness (not all questionnaires were administered to all TBWS participants) was not included. Across all time points (excluding week 3), 94% (2174/2317) of survey responses were complete, 5% (105/2317) included a single missing item, and 2% (38/2317) included multiple missing items. Regarding the shorter survey taken at week 3 that includes the assessment of group memberships, mood, identification with the intervention group, loneliness, and working alliance, 96% (499/518) of responses were complete, 3% (16/518) included a single missing item, and 1% (3/518) included multiple missing items (0‐7 missing items per survey). The level of missingness within given survey responses appears acceptable, especially when considering the length of the longer surveys (55 items, approximately 15‐23 min).

Missingness was more prevalent for the selected background variables (Table 2). Of all survey responses of these selected variables, 79% (581/732) of responses were complete, 19% (141/732) included a single missing item, and 1% (10/732) included multiple missing items. Most often, participants left their number of children unreported. This may be due to a misunderstanding where some childless participants skipped this question instead of reporting zero. However, as the overall missingness in the selected background variables is 3% (172/5856), it is still within acceptable limits (<5%).

## Discussion

### Overview

Psychological ill-being and loneliness are highly prevalent among university students, often leading to long-lasting negative effects on mental health, social connectedness, and academic performance. To address this growing issue, accessible and preventive services at universities are needed. We investigate the effectiveness of the G4H intervention for university students’ well-being using both quantitative and qualitative methods including 2 active comparators.

### Principal Findings

We hypothesize that all the active interventions will improve students’ well-being and that the decrease in loneliness will be more pronounced for the participants of the G4H group, in line with earlier studies [17,24,33,37]. We also anticipate valuable qualitative insights into the experiences of minority students. The multimethod approach will provide a comprehensive understanding of the intervention’s effectiveness and the contextual factors influencing its impact.

### Strengths and Limitations

A key strength of this study is its multifaceted approach, which allows for a comprehensive exploration of existing student well-being interventions at the UH. By involving multiple collaborators, each with unique research interests and expertise, the study can address a wide range of critical questions related to the interventions’ impact. This collaborative framework enables the examination of various mediators and moderators, as well as the integration of both qualitative and quantitative methods. This perspective not only enriches the understanding of students’ well-being but also ensures that these phenomena are not oversimplified. The multimethod design of the study allows participants to express their thoughts and experiences in more depth, enhancing participant engagement and potentially leading to richer data.

Furthermore, the qualitative insights gained from this approach will provide a better understanding of the intervention’s effectiveness—or lack thereof—from the perspective of minority students. This insight is crucial for further developing the G4H intervention in terms of inclusion and equality. Moreover, the study is particularly relevant from an implementation point of view, as it will ensure the suitability and effectiveness of the method in Finland, addressing the significant need for early-stage support within the service system.

This study also has some limitations. The comparator arms (SPGs and TBWS) differ from the G4H slightly in terms of intensity (G4H: 5 sessions, TBWS: 6 sessions, and SPGs: 5‐7 sessions) and delivery methods. This variability complicates direct comparisons between these groups and the G4H intervention, potentially introducing confounding factors that could influence the study’s outcomes. Additionally, the varying data collection methods may introduce mode effects or measurement bias, as well as structural missingness, impacting the reliability and comparability of the data. To mitigate these concerns, we will conduct sensitivity analyses and discuss the implications of these methodological differences in our results.

Furthermore, the lack of exclusion criteria and the nonrandomized design of the protocol may introduce potential biases regarding group composition. However, allowing students to choose which intervention they participate in respects their autonomy and aligns with ethical research practices. This nonrandomized design increases the ecological validity of our findings by capturing the experiences of students in a real-world educational environment, which is important for making informed decisions that can improve services at both individual and population levels [80]. Ecological validity was further increased by including all students in the interventions and in the study. We will implement strategies to address potential selection bias, including collecting detailed demographic and baseline data and using inverse propensity score weighting using double robust estimators, if necessary.

Due to the absence of comparable previous research with the same study population, a priori sample size calculations were not conducted. This represents a limitation, as underpowered samples may fail to detect treatment effects, while overpowered samples may lead to unnecessary use of resources. Nonetheless, the existing literature indicates that significant treatment effects can be observed with smaller sample sizes in different populations [17,24]. Given the complexity of our analysis plan, which includes mixed model analyses and potential (double robust) inverse propensity score weighting, we believe it was justified to aim for a larger target sample size in this study.

We did not appoint an independent data monitoring committee, as this is a naturalistic trial without randomization, and it investigates interventions that focus on psychosocial well-being rather than alleviating psychiatric pathologies. Moreover, there were no clinical inclusion criteria, and as the participants were university students, most showed rather low levels of psychological distress. Therefore, we anticipated minimal risk of harm from the interventions, a decision that was also accepted by the ethical review board of the study. However, we recognize that the presence of such a committee could have enhanced the integrity and trustworthiness of the data collection process.

The study protocol was registered retrospectively. This lack of prospective registration may affect the perceived transparency and credibility of the work. However, in this protocol paper, we have aimed to enhance transparency by providing a comprehensive and detailed description of the study design and methodology before analyzing and publishing the results.

### Future Directions

These results can be expected to be generalizable across Finnish higher education, but caution is needed when extending the results beyond the higher education context or to institutions in other countries. Future studies should investigate the implementation and effectiveness of these interventions in different contexts, including other educational institutions in Finland and internationally, to enhance the understanding of their applicability and impact.

### Dissemination

The trial results will be published in peer-reviewed scientific journals and scientific conferences. We follow the authorship eligibility guidelines of the International Committee of Medical Journal Editors [81]. We do not intend to use professional writers.

### Conclusions

If proven effective, the interventions outlined in this protocol could be implemented in universities nationwide and have a significant impact in improving university students’ well-being in both the short and long term, fostering positive mental health and supporting academic success and future career paths. These models may also be applicable in other settings, such as basic and secondary education, and in workplace environments. Ultimately, this research aims to support inclusion and equality in youth well-being services.

## Supplementary material

10.2196/79319 Multimedia Appendix 1 Details of all questionnaires and scales used in the study, including information on number of items, subscales, scoring, reliability, and validity.

10.2196/79319 Checklist 1 SPIRIT checklist.

## Abbreviations

CBT: cognitive behavioral therapy
ECTS: European Credit Transfer and Accumulation System
EQ-5D-5L: EuroQol - 5 dimension - 5 level questionnaire
G4H: Groups 4 Health
GDPR: General Data Protection Regulation
GPA: grade point average
LGBTIQ+: lesbian, gay, bisexual, transgender, intersex, queer, and other
REDCap: Research Electronic Data Capture
RQ: research question
SIAH: social identity approach to health
SIMIC: social identity model of identity change
SPG: study psychologists’ group intervention
SPIRIT: Standard Protocol Items: Recommendations for Interventional Trials
TBWS: toward better well-being and studying intervention
UH: University of Helsinki

## References

[1] Sheldon E, Simmonds-Buckley M, Bone C (2021). Prevalence and risk factors for mental health problems in university undergraduate students: a systematic review with meta-analysis. J Affect Disord.

[2] Parikka S, Holm N, Koskela T, Ikonen J, Kilpeläinen H (2022). Health and well-being survey of higher education students 2021: research implementation and methods [Article in Finnish]. https://www.julkari.fi/server/api/core/bitstreams/1086b6c3-2eab-4b20-a020-22942305f9c2/content.

[3] Duffy A, Keown-Stoneman C, Goodday S (2020). Predictors of mental health and academic outcomes in first-year university students: identifying prevention and early-intervention targets. BJPsych Open.

[4] Auerbach RP, Mortier P, Bruffaerts R (2018). WHO World Mental Health Surveys International College Student Project: prevalence and distribution of mental disorders. J Abnorm Psychol.

[5] Goosby BJ, Bellatorre A, Walsemann KM, Cheadle JE (2013). Adolescent loneliness and health in early adulthood. Sociol Inq.

[6] Hawkley LC, Cacioppo JT (2010). Loneliness matters: a theoretical and empirical review of consequences and mechanisms. Ann Behav Med.

[7] Holt-Lunstad J, Smith TB, Layton JB (2010). Social relationships and mortality risk: a meta-analytic review. PLoS Med.

[8] Walton GM, Cohen GL (2011). A brief social-belonging intervention improves academic and health outcomes of minority students. Science.

[9] Robbins SB, Oh IS, Le H, Button C (2009). Intervention effects on college performance and retention as mediated by motivational, emotional, and social control factors: integrated meta-analytic path analyses. J Appl Psychol.

[10] French B, Daley D, Groom M, Cassidy S (2023). Risks associated with undiagnosed ADHD and/or autism: a mixed-method systematic review. J Atten Disord.

[11] Kieseppä V, Holm M, Jokela M, Suvisaari J, Gissler M, Lehti V (2021). Depression and anxiety disorders among immigrants living in Finland: comorbidity and mental health service use. J Affect Disord.

[12] Lehtonen J, Majlander S, Sares-Jäske L, Jehkoi A, Luopa P (2024). Well-being of 8th and 9th graders belonging to gender and sexual minorities – school health survey 2019–2023 [Article in Finnish]. https://www.julkari.fi/server/api/core/bitstreams/d4d0fbb3-e6c9-494d-a86a-4f524409a6c8/content.

[13] Settles IH, Buchanan NT (2014). The Oxford Handbook of Multicultural Identity.

[14] Vargas SM, Huey SJ, Miranda J (2020). A critical review of current evidence on multiple types of discrimination and mental health. Am J Orthopsychiatry.

[15] Peltola M, Moisio J (2017). Voices and silence in service fields: a review of information on children and young people’s service experiences [Article in Finnish]. https://edition.fi/nuorisotutkimusseura/catalog/view/300/236/814.

[16] Green AE, Price MN, Dorison SH (2022). Cumulative minority stress and suicide risk among LGBTQ youth. Am J Community Psychol.

[17] Haslam C, Cruwys T, Haslam SA, Dingle G, Chang MXL (2016). Groups 4 Health: evidence that a social-identity intervention that builds and strengthens social group membership improves mental health. J Affect Disord.

[18] Haslam C, Jetten J, Cruwys T, Dingle GA, Haslam SA (2018). The New Psychology of Health.

[19] Haslam SA, Jetten J, Postmes T, Haslam C (2009). Social identity, health and well‐being: an emerging agenda for applied psychology. Applied Psychology.

[20] Iyer A, Jetten J, Tsivrikos D (2008). Self Continuity: Individual and Collective Perspectives.

[21] Haslam C, Haslam SA, Jetten J, Cruwys T, Steffens NK (2021). Life change, social identity, and health. Annu Rev Psychol.

[22] Tajfel H, Tirner J, Worchel S, Austin WG (1979). The Social Psychology of Intergroup Relations.

[23] Turner JC (1987). Rediscovering the Social Group: A Self-Categorization Theory.

[24] Cruwys T, Haslam C, Rathbone JA, Williams E, Haslam SA, Walter ZC (2022). Groups 4 Health versus cognitive-behavioural therapy for depression and loneliness in young people: randomised phase 3 non-inferiority trial with 12-month follow-up. Br J Psychiatry.

[25] Baier AL, Kline AC, Feeny NC (2020). Therapeutic alliance as a mediator of change: a systematic review and evaluation of research. Clin Psychol Rev.

[26] Gergov V, Marttunen M, Lindberg N, Lipsanen J, Lahti J (2021). Therapeutic alliance: a comparison study between adolescent patients and their therapists. Int J Environ Res Public Health.

[27] Delgadillo J, Branson A, Kellett S, Myles-Hooton P, Hardy GE, Shafran R (2020). Therapist personality traits as predictors of psychological treatment outcomes. Psychother Res.

[28] Heinonen E, Lindfors O, Laaksonen MA, Knekt P (2012). Therapists’ professional and personal characteristics as predictors of outcome in short- and long-term psychotherapy. J Affect Disord.

[29] Heinonen E, Lindfors O, Härkänen T, Virtala E, Jääskeläinen T, Knekt P (2014). Therapists’ professional and personal characteristics as predictors of working alliance in short-term and long-term psychotherapies. Clin Psychol Psychother.

[30] Ryan RM, Deci EL (2017). Self-Determination Theory: Basic Psychological Needs in Motivation, Development, and Wellness.

[31] Drieschner KH, Lammers SMM, van der Staak CPF (2004). Treatment motivation: an attempt for clarification of an ambiguous concept. Clin Psychol Rev.

[32] Haslam C, Cruwys T, Bentley S, Haslam AS, Dingle GA, Jetten J (2021). GROUPS 4 HEALTH Facilitator Manual (Version 40).

[33] Haslam C, Cruwys T, Chang MXL (2019). GROUPS 4 HEALTH reduces loneliness and social anxiety in adults with psychological distress: findings from a randomized controlled trial. J Consult Clin Psychol.

[34] Asikainen H, Katajavuori N (2021). Development of a web-based intervention course to promote students’ well-being and studying in universities: protocol for an experimental study design. JMIR Res Protoc.

[35] Hayes SC, Luoma JB, Bond FW, Masuda A, Lillis J (2006). Acceptance and commitment therapy: model, processes and outcomes. Behav Res Ther.

[36] Howell AJ, Passmore HA (2019). Acceptance and commitment training (ACT) as a positive psychological intervention: a systematic review and initial meta-analysis regarding ACT’s role in well-being promotion among university students. J Happiness Stud.

[37] Katajavuori N, Vehkalahti K, Asikainen H (2023). Promoting university students’ well-being and studying with an acceptance and commitment therapy (ACT)-based intervention. Curr Psychol.

[38] Räihä K, Katajavuori N, Vehkalahti K, Huotilainen M, Asikainen H (2024). University students’ stress and burnout risk: results of an ACT-based online-course using self-assessments and HRV-measurements. Curr Psychol.

[39] Faul F, Erdfelder E, Lang AG, Buchner A (2007). G*Power 3: a flexible statistical power analysis program for the social, behavioral, and biomedical sciences. Behav Res Methods.

[40] Cohen J (1988). Statistical Power Analysis for the Behavioral Sciences.

[41] Harris PA, Taylor R, Thielke R, Payne J, Gonzalez N, Conde JG (2009). Research Electronic Data Capture (REDCap)--a metadata-driven methodology and workflow process for providing translational research informatics support. J Biomed Inform.

[42] Hays RD, DiMatteo MR (1987). A short-form measure of loneliness. J Pers Assess.

[43] Russell D, Peplau LA, Cutrona CE (1980). The revised UCLA Loneliness Scale: concurrent and discriminant validity evidence. J Pers Soc Psychol.

[44] Kroenke K, Spitzer RL, Williams JBW (2001). The PHQ-9: validity of a brief depression severity measure. J Gen Intern Med.

[45] Haslam C, Holme A, Haslam SA, Iyer A, Jetten J, Williams WH (2008). Maintaining group memberships: social identity continuity predicts well-being after stroke. Neuropsychol Rehabil.

[46] Shah N, Cader M, Andrews B, McCabe R, Stewart-Brown SL (2021). Short Warwick-Edinburgh Mental Well-being Scale (SWEMWBS): performance in a clinical sample in relation to PHQ-9 and GAD-7. Health Qual Life Outcomes.

[47] Evans C, Connell J, Audin K, Sinclair A, Barkham M (2005). Rationale and development of a general population well-being measure: psychometric status of the GP-CORE in a student sample. Br J Guid Counc.

[48] Spitzer RL, Kroenke K, Williams JBW, Löwe B (2006). A brief measure for assessing generalized anxiety disorder: the GAD-7. Arch Intern Med.

[49] Connor KM, Kobak KA, Churchill LE, Katzelnick D, Davidson JRT (2001). Mini-SPIN: a brief screening assessment for generalized social anxiety disorder. Depress Anxiety.

[50] The EuroQol Group (1990). EuroQol - a new facility for the measurement of health-related quality of life. Health Policy.

[51] Postmes T, Haslam SA, Jans L (2013). A single‐item measure of social identification: reliability, validity, and utility. British J Social Psychol.

[52] Hatcher RL, Gillaspy JA (2006). Development and validation of a revised short version of the Working Alliance Inventory. Psychother Res.

[53] Hatcher RL, Lindqvist K, Falkenström F (2020). Psychometric evaluation of the Working Alliance Inventory-Therapist version: current and new short forms. Psychother Res.

[54] Sarason IG, Sarason BR, Shearin EN, Pierce GR (1987). A brief measure of social support: practical and theoretical implications. J Soc Pers Relat.

[55] Guay F, Vallerand RJ, Blanchard C (2000). On the assessment of situational intrinsic and extrinsic motivation: the Situational Motivation Scale (SIMS). Motiv Emot.

[56] Miller WR, Rollnick S (2012). Motivational Interviewing: Helping People Change.

[57] Parpala A, Lindblom-Ylänne S (2012). Using a research instrument for developing quality at the university. Quality in Higher Education.

[58] Konstabel K, Lönnqvist JE, Leikas S (2017). Measuring single constructs by single items: constructing an even shorter version of the “Short Five” personality inventory. PLoS ONE.

[59] Wei M, Russell DW, Mallinckrodt B, Vogel DL (2007). The Experiences in Close Relationship Scale (ECR)-short form: reliability, validity, and factor structure. J Pers Assess.

[60] Heinonen E, Orlinsky DE, Willutzki U (2022). Psychotherapist trainees’ quality of life: patterns and correlates. Front Psychol.

[61] Hartmann A, Orlinsky DE, Rønnestad MH, Willutzki U, Schröder TA, Heinonen E (2025). Measuring psychotherapist functioning with the Therapist Work Involvement Scales (TWIS): reliability, factor structure, and measurement invariance. Psychother Res.

[62] Mäklin S, Kokko P (2021). Unit costs of health and social care in Finland in 2017 [Article in Finnish]. https://www.julkari.fi/server/api/core/bitstreams/5268c80a-cf96-473b-b525-679e7bf02905/content.

[63] van Buuren S, Groothuis-Oudshoorn K (2011). mice: multivariate imputation by chained equations in R. J Stat Softw.

[64] Kurz CF (2022). Augmented inverse probability weighting and the double robustness property. Med Decis Making.

[65] Wager S (2025). Causal Inference: A Statistical Learning Approach.

[66] Galwey NW (2009). A new measure of the effective number of tests, a practical tool for comparing families of non-independent significance tests. Genet Epidemiol.

[67] Sun S, Chuang LH, Sahlén KG, Lindholm L, Norström F (2022). Estimating a social value set for EQ-5D-5L in Sweden. Health Qual Life Outcomes.

[68] Faria R, Gomes M, Epstein D, White IR (2014). A guide to handling missing data in cost-effectiveness analysis conducted within randomised controlled trials. Pharmacoeconomics.

[69] Hoch JS, Briggs AH, Willan AR (2002). Something old, something new, something borrowed, something blue: a framework for the marriage of health econometrics and cost-effectiveness analysis. Health Econ.

[70] Fraser G, Brady A, Wilson MS (2022). Mental health support experiences of Rainbow Rangatahi youth in Aotearoa New Zealand: results from a co-designed online survey. J R Soc N Z.

[71] McDermott E, Eastham R, Hughes E (2024). “What Works” to support LGBTQ+ young people’s mental health: an intersectional youth rights approach. Int J Soc Determinants Health Health Serv.

[72] Mailander S, Lehtonen J, Luopa P, Kekkonen M, Gissler M, Känkänen P, Isola AM (2022). Exceptional Youth in the Time of Corona: Youth Living Conditions Yearbook 2022 [Book in Finnish].

[73] Nori H, Lyytinen A, Juusola H, Kohtamäki V, Kivistö J (2021). Marginal groups in higher education: applying to study, study experiences and future plans [Article in Finnish]. https://julkaisut.valtioneuvosto.fi/server/api/core/bitstreams/b73fe053-dd66-4c57-968d-908b38a6708b/content.

[74] (2022). Values and attitudes of University of Applied Students 2022 [Article in Finnish]. https://samok.fi/wp-content/uploads/2022/11/samok_arvot-ja-asenteet.pdf.

[75] Meyer IH (2003). Prejudice, social stress, and mental health in lesbian, gay, and bisexual populations: conceptual issues and research evidence. Psychol Bull.

[76] Collins PH (2019). Intersectionality as Critical Social Theory.

[77] Moradi B, Grzanka PR (2017). Using intersectionality responsibly: toward critical epistemology, structural analysis, and social justice activism. J Couns Psychol.

[78] Braun V, Clarke V (2006). Using thematic analysis in psychology. Qual Res Psychol.

[79] Braun V, Clarke V (2021). Thematic analysis: a practical guide.

[80] Singal AG, Higgins PDR, Waljee AK (2014). A primer on effectiveness and efficacy trials. Clin Transl Gastroenterol.

[81] Defining the role of authors and contributors. International Committee of Medical Journal Editors.

